# Validating an algorithm to identify metastatic gastric cancer in the absence of routinely collected TNM staging data

**DOI:** 10.1186/s12913-018-3125-7

**Published:** 2018-05-02

**Authors:** Alyson L. Mahar, Yunni Jeong, Brandon Zagorski, Natalie Coburn

**Affiliations:** 1 Community Health Sciences, University of Manitoba, Winnipeg MB; 727 McDermot Ave, Winnipeg, MB R3P 3P5 Canada; 2 Division of General Surgery, Department of Surgery & Institute of Health Policy, Management and Evaluation, University of Toronto, K3W-15, Sunnybrook Health Sciences Centre 2075 Bayview Avenue, Toronto, ON M4N 3M5 Canada; 3 Institute of Health Policy, Management, and Evaluation, University of Toronto, 4th Floor, 155 College St, Toronto, ON M5T 3M6 Canada; 4 Department of Surgery & Institute of Health Policy, Management, and Evaluation, University of Toronto, T2-11, Odette Cancer Centre, 2075 Bayview Avenue, Toronto, ON M4N 3M5 Canada

**Keywords:** Gastric adenocarcinoma, Metastatic disease, Staging, Algorithm

## Abstract

**Background:**

Accurate TNM stage information is essential for cancer health services research, but is often impractical and expensive to collect at the population-level. We evaluated algorithms using administrative healthcare data to identify patients with metastatic gastric cancer.

**Methods:**

A population-based cohort of gastric cancer patients diagnosed between 2005 and 2007 identified from the Ontario Cancer Registry were linked to routinely collected healthcare data. Reference standard data identifying metastatic disease were obtained from a province-wide chart review, according to the Collaborative Staging method. Algorithms to identify metastatic gastric cancer were created using administrative healthcare data from hospitalization, emergency department, and physician billing records. Time frames of data collection in the peri-diagnosis period, and the diagnosis codes used to identify metastatic disease were varied. Algorithm sensitivity, specificity, and accuracy were evaluated.

**Results:**

Of 2366 gastric cancer patients, included within the chart review, 54.3% had metastatic disease. Algorithm sensitivity ranged from 50.0- 90%, specificity ranged from 27.6 - 92.5%, and accuracy from 61.5 - 73.4%. Sensitivity and specificity were maximized when the most conservative list of diagnosis codes from hospitalization and outpatient records in the six months prior to and the six months following diagnosis were included.

**Conclusion:**

Algorithms identifying metastatic gastric cancer can be used for research purposes using administrative healthcare data, although they are imperfect measures. The properties of these algorithms may be generalizable to other high fatality cancers and other healthcare systems. This study provides further support for the collection of population-based, TNM stage data.

**Electronic supplementary material:**

The online version of this article (10.1186/s12913-018-3125-7) contains supplementary material, which is available to authorized users.

## Background

Stage data is needed to define clinically homogenous cohorts, adjust for the extent of disease spread, study real-world treatment effectiveness and costs, and inform regional decision-making [1]. Accurate staging, when linked to treatment and outcome data, informs the effectiveness and quality of cancer treatments, and guides healthcare planning for resource mobilization or implementation [1]. The absence of stage data increases the complexity of maintaining representativeness of the cancer cohort, minimizing bias caused by excluding patients with unknown stage data, and achieving adequate sample size to perform robust statistical analyses [2].

Capturing population-based stage data in ‘big data’ is often limited by practical and financial constraints. For example, the International Cancer Benchmarking Project used multiple national cancer registries to understand cancer stage and survival patterns [3]. The registries contained varying levels of complete stage data across primary cancer sites; upwards of 50% of patients were excluded due to missing stage data in this international comparison of cancer survival [4, 5]. As a result, many countries are aiming to improve their population-based stage data collection using a number of methods and data sources [1, 2, 6–8].

Validated algorithms to identify metastatic disease using routinely collected healthcare data may provide one solution to missing stage data in studies using population-based, administrative data [9, 10]. Benchimol et al. have published general guidelines for algorithm development and validation using administrative healthcare data to assign disease status [9]. Overall, many studies do not appropriate report on the performance of the algorithm, including revalidation, present at least four metrics to assess diagnostic accuracy (e.g. sensitivity, specificity, agreement), or confidence intervals [9]. Little published research has evaluated algorithm performance across cancer sites; developing high quality algorithms requires gold standard staging data to properly validate and ensure accuracy prior to use. Whyte et al. evaluated 28 algorithms to identify metastatic disease status in three administrative data cohorts of treated colorectal, breast, and lung cancer patients in the United States [11]. The algorithms had varying properties depending on cancer site, the underlying prevalence of metastatic disease, the choice of timeframe, and diagnosis codes [11]. This is consistent with the properties of other diagnostic algorithms, where there is also evidence that algorithm performance is dependent on the data sources used.

Gastric cancer (GC) is the third leading cause of cancer-related mortality worldwide [12, 13]. Most patients in North America present with metastatic disease at diagnosis [14, 15], with similar stage distributions reported in the United Kingdom [16–18]. Although not all countries capture this information routinely, the ability to identify stage IV patients in population-based registries is crucial. Therefore, this study linked detailed TNM staging data from a province-wide chart review with routinely collected healthcare data, to develop an algorithm to identify individuals with metastatic disease in a cohort of GC patients.

## Methods

### Study population

GC patients aged 19 and older and diagnosed between April 1, 2005 and March 31, 2008 were identified in the Ontario Cancer Registry. Patients with multiple cancers, no corresponding hospital chart, tumour located primarily in the oesophagus, or non-adenocarcinoma tumours were excluded. The project received the Research Ethics Boards approval at the Sunnybrook Health Sciences Centre and adhered to all privacy and confidentiality regulations of ICES. Individual patient consent was not required. ICES is an s. Forty five Prescribed Entity under Ontario’s privacy law (PHIPA), enabling us to study the health and health outcomes of individuals for the purpose of analysis or compiling statistical information with respect to the management of, evaluation or monitoring of, the allocation of resources to or planning for all or part of the health system.

### Data sources

A province-wide chart review was conducted at over 100 institutions between November 2009 and November 2011. Information from multiple endoscopy, radiology, and pathology reports per patient were aggregated. Data abstraction from operative reports was completed by a surgical resident in 2013. Chart review data were linked to routinely collected healthcare and vital status data at ICES in 2013. All hospitalizations, emergency department (ED) visits, and physician visits were captured from the Canadian Institute of Health Information-Discharge Abstract Database and the Same Day Surgery Database, the National Ambulatory Care Reporting System, and the Ontario Health Insurance Plan database.

### Metastatic disease

#### Reference standard

The 7th Edition American Joint Committee on Cancer/Union International Cancer Control TNM staging system was used [19]. TNM stage data from patient hospital charts were used as the reference standard. Stage data were collected in the 180 days prior to the diagnosis date registered in the Ontario Cancer Registry and in the 180 days following diagnosis up until the date of surgical resection (whichever came last) using a modified Collaborative Staging system approach. Clinic, diagnostic imaging, endoscopy, surgery, and pathology records were used to identify metastatic disease. Patients were considered stage IV, otherwise defined as M1 or positive for metastatic disease, if evidence of metastatic disease was identified in any portion of the medical record and M0 otherwise (stage I-III).

#### Algorithms

Three sets of administrative data algorithms to identify stage IV gastric cancer [19], otherwise defined as the presence of metastatic disease at diagnosis, were created using a combination of information from hospitalization records, ED visits, and outpatient physician visits. A positive diagnosis of metastatic GC was determined using three sets of eligible International Classification of Disease (ICD) system version 9 and 10 diagnosis codes (a complete list is provided in Additional file 1: Table S1). The included diagnoses ranged from conservative (secondary malignancy codes only, e.g. ICD-9 code 196) to inclusive (any non-gastric malignancy diagnosis (e.g., ICD 10 C codes excluding digestive organs). In the first set of algorithms, patients were identified as being metastatic if they had a hospitalization. In the second set of algorithms, patients with metastases were identified using hospitalization records (one or more) and outpatient records (two or more). In the third set of algorithms, patients with metastases were identified if they had one or more hospitalizations or outpatient records. Three different time periods were also considered for each algorithm: three months pre- and post-diagnosis, six months pre- and post-diagnosis, and three months pre-diagnosis with no end to follow-up post-diagnosis. These specific criteria were chosen based on the types of data in our administrative data holdings, as well as previous studies defining metastatic disease using similar data, and based on the properties of diagnostic algorithms using administrative data in other settings. We performed a sensitivity analysis restricting the cohort to those who received a surgical resection. In total, 45 algorithms were evaluated.

### Statistical analysis

Sensitivity, specificity, positive predictive value, negative predictive value, and accuracy were calculated for each algorithm. Accuracy was measured using the following equation: Accuracy = (TP + TN) / (TP + TN + FP + FN) [9]. Ninety five percent confidence limits on the estimates of sensitivity, specificity, PPV, NPV and accuracy were calculated using percentiles of a distribution of 5000 bootstrap replicates with replacement. Demographic characteristics and the tumour stage, lymph node status, and TNM stage of true positives, false positives, true negatives, and false negatives were described for each algorithm. Content validity was evaluated by comparing the percentage of patients who died in year following diagnosis.

## Results

Overall, 2366 patients were included; 54.3% had metastasis at diagnosis according to the chart review (Table 1). Sensitivity, specificity, and accuracy of the algorithms are reported in Table 2. Sensitivity ranged from 50.0 - 90%, specificity ranged from 27.6 - 92.5%, and accuracy from 61.5 - 73.4%. Sensitivity and specificity were maximized when the algorithm used the most conservative list of metastatic disease diagnosis codes, hospitalization and outpatient records as the data source, and when the algorithm was run on administrative data from the six months prior to and following diagnosis. The sensitivity of the algorithms all decreased and the specificity of the algorithms increased slightly, when the cohort was restricted to patients who received surgical resection (Additional file 2: Table S2). Excluding patients with unknown metastatic disease status (4.3%) did not change the results (data not shown). Concordant and discordant classifications between the algorithms and the reference standard are reported in Additional file 3: Table S3.

**Table 1:** A description of the reference standard M1 and non-M1 cohort

Characteristic	Reference Standard M1	
	1 (*n* = 1285)	0 (*n* = 1081)
Age		
< 50	10.9	6.8
50–54	6.9	5.9
55–59	8.9	8.6
60–64	10.4	10.5
65–69	13.3	11.8
70+	49.6	56.2
Female	35	35.5
Charlson Category		
No Prev Hosp	56.9	47.8
0	28.6	30.6
1	12.5	4.7
2+	6.9	11
Tumour Location		
Distal	32.9	43.8
Entire	9.8	5.1
GEJ	27.3	23.5
Middle	15.6	17.1
Proximal	9.8	6.9
Unknown	4.7	3.6
Deaths		
One year	71.9	32.5
Five years	94.6	64.7

**Table 2:** Properties of evaluated algorithms

Algorithm	Se (95% CI)	Sp (95% CI)	PPV (95% CI)	NPV (95% CI)	A (95% CI)
+/− 3 Months Diagnosis Date					
Conservative Diagnosis Codes					
1. 1+ hospitalization	50 (47.2–52.8)	92.5 (90.9–94.0)	88.8 (86.4–91.0)	60.9 (58.5–63.3)	69.4 (67.6–71.3)
2. 1+ hospitalization or 2+ outpatient	57.8 (55.1–60.5)	86.9 (84.8–88.8)	84 (81.4–86.3)	63.4 (60.9–65.9)	71 (69.2–72.9)
3. 1+ hospitalization or 1+ outpatient	61.2 (58.5–63.9)	84.2 (82.0–86.3)	82.1 (79.6–84.6)	64.6 (62.1–67.1)	71.7 (69.8–73.5)
Less Conservative Diagnosis Codes					
4. 1+ hospitalization	52.9 (50.2–55.7)	87.9 (85.9–89.8)	83.8 (81.2–86.3)	61.1 (58.6–63.5)	68.9 (67.0–70.8)
5. 1+ hospitalization or 2+ outpatient	60.4 (57.7–63.2)	82.2 (79.9–84.4)	80.1 (77.6–82.6)	63.6 (61.0–66.1)	70.3 (68.5–72.1)
6. 1+ hospitalization or 1+ outpatient	64.2 (61.5–66.8)	79.7 (77.4–82.2)	79 (76.5–81.4)	65.2 (62.5–67.8)	71.3 (69.5–73.1)
Most Inclusive Diagnosis Codes					
7. 1+ hospitalization	53.6 (50.9–56.4)	86.9 (84.8–88.9)	82.9 (80.3–85.5)	61.2 (58.7–63.7)	68.8 (66.9–70.7)
8. 1+ hospitalization or 2+ outpatient	69.5 (66.9–72.0)	68 (65.2–70.8)	72.1 (69.4–74.5)	65.2 (62.4–68.0)	68.8 (66.9–70.6)
9. 1+ hospitalization or 1+ outpatient	76.3 (73.9–78.6)	58.7 (55.8–61.7)	68.7 (66.2–71.1)	67.5 (64.4–70.5)	68.2 (66.3–70.1)
+/− 6 months Diagnosis Date					
Conservative Diagnosis Codes					
10. 1+ hospitalization	57.5 (54.7–60.3)	90.1 (88.3–91.8)	87.4 (85.0–89.5)	64.1 (61.7–66.6)	72.4 (70.6–74.2)
11. 1+ hospitalization or 2+ outpatient	65.8 (63.2–68.4)	82.3 (80.0–84.6)	81.6 (79.2–83.9)	66.9 (64.4–69.5)	73.3 (71.6–75.1)
12. 1+ hospitalization or 1+ outpatient	68.6 (66.1–71.2)	79.1 (76.7–81.6)	79.6 (77.2–82.0)	67.9 (65.3–70.5)	73.4 (71.6–75.1)
Less Conservative Diagnosis Codes					
13. 1+ hospitalization	60.1 (57.4)	84.4 (82.2–86.5)	82.1 (79.6–84.5)	64 (61.5–66.5)	71.2 (69.4–73.0)
14. 1+ hospitalization or 2+ outpatient	68.1 (65.6–70.6)	77.1 (74.5–79.6)	77.9 (75.4–80.3)	67 (64.4–69.7)	72.2 (70.4–74.0)
15. 1+ hospitalization or 1+ outpatient	71 (68.6–73.5)	73.8 (71.2–76.4)	76.3 (73.9–78.7)	68.2 (65.5–70.9)	72.3 (70.5–74.0)
Most Inclusive Diagnosis Codes					
16. 1+ hospitalization	61.1 (58.4–63.8)	83 (80.7–85.2)	81 (78.5–83.4)	64.2 (61.7–66.8)	71.1 (69.3–73.0)
17. 1+ hospitalization or 2+ outpatient	77.4 (75.1–79.7)	57.8 (54.8–60.6)	68.5 (66.0–70.9)	68.2 (65.3–71.3)	68.4 (66.6–70.2)
18. 1+ hospitalization or 1+ outpatient	82.7 (80.6–84.8)	48.2 (45.2–51.2	65.5 (63.1–67.8)	70.1 (66.7–73.5)	66.9 (65.0–68.9)
−3 months Diagnosis, No Time Limit					
Conservative Diagnosis Codes					
19. 1+ hospitalization	70.7 (68.1–73.2)	69.9 (67.1–72.6)	73.6 (71.0–76.0)	66.7 (63.9–69.5)	70.3 (68.4–72.1)
20. 1+ hospitalization or 2+ outpatient	77.4 (75.1–79.6)	60.4 (57.5–63.3)	69.9 (67.5–72.3)	69.2 (66.2–72.2)	69.6 (67.8–71.5)
21. 1+ hospitalization or 1+ outpatient	80.2 (78.0–82.4)	55.6 (52.7–58.6)	68.2 (65.8–70.6)	70.2 (67.1–73.3)	68.9 (67.1–70.9)
Less Conservative Diagnosis Codes					
22. 1+ hospitalization	72.9 (70.5–75.3)	63.9 (61.1–66.8)	70.6 (68.1–72.9)	66.6 (63.6–69.4)	68.8 (66.9–70.6)
23. 1+ hospitalization or 2+ outpatient	79.3 (77.1–81.5)	55 (52.1–58.0)	67.7 (65.3–70.0)	69.1 (66.0–72.2)	68.3 (66.4–70.1)
24. 1+ hospitalization or 1+ outpatient	82.2 (80.1–84.3)	50.4 (47.5–53.4)	66.3 (64.0–68.6)	70.4 (67.2–73.7)	67.7 (65.8–69.6)
Most Inclusive Diagnosis Codes					
25. 1+ hospitalization	74 (71.6–76.4)	62 (59.2–64.9)	69.8 (67.4–72.2)	66.8 (63.9–69.7)	68.6 (66.6–70.4)
26. 1+ hospitalization or 2+ outpatient	86.5 (84.7–88.4)	35.3 (32.5–38.2)	61.4 (59.1–63.6)	68.8 (65.1–72.6)	63.2 (61.2–65.1)
27. 1+ hospitalization or 1+ outpatient	90.1 (88.4–91.7)	27.6 (24.9–30.2)	59.7 (57.4–61.8)	70 (65.7–74.4)	61.5 (59.6–63.5)

*95% CI* bootstrapped 95% confidence intervals, *Se* sensitivity, *Sp* specificity, *PPV* positive predictive value, *NPV* negative predictive value, *A* accuracy

Table 3 describes the algorithm that maximized sensitivity and specificity (algorithm # 12). According to this algorithm, the prevalence of metastatic GC was 45%. Of the 1285 true positives using the reference standard, 31% were misclassified using this administrative healthcare data algorithm; 20% of the metastatic group identified by the algorithm were false positives and 32% of the M0 were false negatives. One third of the false positives and false negatives had an unknown stage at diagnosis according to the reference standard. Correctly classified metastatic patients were more likely to have died within a year of diagnosis, than those incorrectly classified.

**Table 3:** A description of concordant and discordant classifications for algorithm 12, the algorithm that maximized sensitivity and specificity

Variable	M1 (*n* = 1107)		M_not_ (*n* = 1259)	
	+/+	+/−	−/+	−/−
	80%	20%	32%	68%
Age (years)				
< 50	12.5	10.6	7.4	5.8
50–54	7.6	8.8	5.4	5.1
55–59	8.9	8.8	9.2	8.5
60–64	11.1	10.2	8.7	10.6
65–69	13.2	11.9	13.6	11.8
70-high	46.8	49.6	55.7	58
Female	35.9	39.4	33.2	34.5
Tumour Location				
Distal	30.4	37.2	37.6	37.7
Entire	9.9	5.8	9.7	7.7
GEJ	27.4	30.5	27.2	25.6
Middle	17	14.2	12.6	16.3
Proximal	10.6	6.6	8.2	8.5
Unknown	4.8	5.8	4.7	4.2
Charlson Score				
No Prev Hosp	56.4	49.6	57.9	47.4
0	29.7	33.2	26.2	29.9
1	7.6	8.8	7.7	11
2+	6.2	8.4	12.9	10
Urban Residence	89.2	89.4	87.6	87.7
Community Income				
Lowest	20.2	23	22.5	20
2	22.9	17.7	23.3	21.6
3	20	23	18.3	20
4	20.3	17.7	16.6	20.1
Highest	16.5	18.6	19.3	17.9
T stage				
Tis/T0/T1	1	4.8	2.4	16
T2	1	7.1	3.2	13.8
T3	3.2	20.8	7.7	15.3
T4A	6.2	22.6	15.6	18.9
T4B	27.7	20.8	26.7	11.6
T1-T4A	4	1.8	4.5	3.3
TX	56.9	22.1	39.9	21.2
N status				
N0	18.2	25.2	21.3	38.6
N1	3.2	11.1	6.7	14.3
N2	4.1	20.4	8.4	13
N3A	6.2	14.6	9.9	10.8
N3B	3.2	5.8	5.9	4.4
N1–3	50.7	11.1	34.6	7.5
NX	15.3	11.9	13.1	11.5
AJCC Stage				
0/IA	0	2.7	0	11.9
IB	0	4	0	9.8
IIA	0	7.5	0	8.9
IIB	0	8	0	8.2
IIIA	0	9.7	0	8.5
IIIB	0	17.7	0	12.2
IIIC	0	17.7	0	11.6
IV	100	0	100	0
Unknown	0	32.3	0	29.2
Death within 1 year	79.7	42.9	55	29.7
Death within 5 years	96.9	74.3	89.6	62.1

+/+ = true positive, +/− false positive, −/+ false negative, −/− true negative

Using the algorithm with the highest positive predictive value (algorithm # 1), 11% of those identified as having metastatic disease were misclassified. Ninety percent of patients misclassified using this algorithm were stage III (55.5%) or unknown stage (34.6%), 66% had a T4a or T4b tumour. Overall, as the positive predictive value of the algorithm decreased, the proportion of node-negative patients with smaller tumours, and earlier stage disease, misclassified as metastatic increased (data not shown).

## Discussion

This study evaluated 45 algorithms using routinely collected healthcare data to identify metastatic disease in a population-based cohort of GC patients. None of the algorithms did an excellent job of classifying patients based on the reference standard. The algorithm that maximized sensitivity and specificity identified metastatic disease through one or more hospitalization or outpatient records with a diagnosis from the conservative list, in the six months before and after diagnosis.

Our algorithm accuracy differed from the few others present in the literature as the result of study design or the underlying prevalence of metastatic disease. We observed lower accuracy than a study of colorectal cancer algorithms by Brooks et al. [20]. Whyte et al. reported better accuracy for their algorithms identifying metastatic disease in breast cancer, and similar accuracy for algorithms in lung and colorectal cancer [11]. Whyte et al. reported sensitivity and specificity estimates ranging from 46 to 77 and 83–99% for breast cancer, 50–67 and 68–83% for lung cancer, and 54–77 and 70–91% for colorectal cancer [11]. Whyte et al. did not define the length of their follow-up period, or explain why the total number of patients varied across algorithms, and only included patients treated within a private healthcare system [11]. Both Whyte et al. and Brooks et al. studied only patients who received treatment. Both breast and colorectal cancer have a much lower prevalence of metastatic disease at diagnosis, compared to GC which may impact accuracy. We concluded similar findings to an algorithm developed by Lash et al. to identify colorectal cancer recurrence, in which patients correctly identified by the algorithm were more likely to be younger and to die in a shorter timeframe [21].

The best algorithm choice is dependent on the research purpose [22]. For example, maximizing accuracy may be the priority when estimating the prevalence of metastatic disease, when representativeness of the identified cohort is not important. Maximizing specificity may be the priority to ensure patients included in a study of metastatic patients are not metastatic. We recommend using a conservative approach with relevant diagnosis codes reported close to the diagnosis date. This approach, and the other algorithms reported in this study should be tested in an additional, external cohort, including one that better reflects current clinical populations and treatment. The properties of algorithms in this study may be generalizable to similar high fatality cancer cohorts such as pancreas and esophagus. The algorithms may be used by other investigators and policy-makers to estimate the extent of misclassification, and in formal bias analyses to adjust effect estimates [23]. Alternatively, given that none of the algorithms demonstrated exemplary accuracy, integrating multiple algorithms using methods such as majority vote and Boolean operations may be another way these algorithms may be implemented in practice [24].

Our study is limited by our choice of a reference standard, which may have resulted in misclassification of metastatic disease across patients. The prevalence of metastatic disease was 54% in our study, with a median survival of six months, which matches the literature distribution [14, 25]. We performed a sensitivity analysis restricting to the cohort of patients with a surgical resection, who would have better quality pathologic staging data available in their charts. The true prevalence of metastatic disease was lower and the positive predictive value of the algorithms decreased. We also attempted to address administrative data quality issues by creating three sets of algorithms based on the data reliability (hospitalization data being most reliable) and using three sets of diagnosis codes.

## Conclusions

We suggest that algorithms using administrative healthcare data are imperfect replacements for population-based staging data and support the need for system level data collection. However, they do yield moderately accurate results. In cases where population-based data collection is infeasible, a global understanding of misclassified patients and administrative algorithm properties is important to assessing potential selection bias.

## Additional files

Additional file 1: **Table S1.** Included diagnoses, ICD-9 and 10 codes used to identify metastatic disease for the different algorithms. (DOCX 15 kb)

Additional file 2: **Table S2.** Algorithm properties when the patient cohort was restricted to those who received a surgical resection, Sensitivity, specificity, negative predictive value, positive predictive value and accuracy for the algorithms when applied to a subset of patients who received a surgical resection. (DOCX 15 kb)

Additional file 3: **Table S3.** Number of patients in each cell, by algorithm, The breakdown of the number of true positive, true negatives, false positives and false negatives for each algorithm. (DOCX 19 kb)

## Abbreviations

ED: Emergency department
FN: False negative
FP: False positive
GC: Gastric cancer
ICD: International Classification of Disease
OCR: Ontario Cancer Registry
PHIPA: Personal Health Information Privacy Act
TN: True negative
TNM: Tumour, node, metastasis
TP: True positive
