# Supplementary material for: Validating an algorithm to identify metastatic gastric cancer in the absence of routinely collected TNM staging data
Source: BMC Health Serv Res. 2018 May 2;18:309. doi: 10.1186/s12913-018-3125-7 (PMC5930789; doi:10.1186/s12913-018-3125-7)
Supplement: Supplementary file 1 — Table S1. Included diagnoses, ICD-9 and 10 codes used to identify metastatic disease for the different algorithms. (DOCX 15 kb) [file 12913_2018_3125_MOESM1_ESM.docx]

| **Table S1: Included diagnoses** |
| --- |
| **Diagnosis List One: Most Conservative** |
| **ICD-9** |
| 196 (except 1962): secondary and unspecified malignant neoplasm of lymph nodes |
| 197 (except 1975, 1978): secondary malignant neoplasm of respiratory and digestive systems |
| 197.5: secondary malignant neoplasm of large intestine and rectum |
| 197.8: secondary malignant neoplasm of other digestive organs and spleen |
| 198: secondary malignant neoplasm of other specified sites |
| **ICD-10** |
| C77 (except C772): secondary and unspecified malignant neoplasm of lymph nodes |
| C77.2: secondary and unspecified malignant neoplasm of intra-abdominal lymph nodes |
| C78: secondary malignant neoplasm of respiratory and digestive organs |
| C79: secondary malignant neoplasm of other and unspecified sites |
| C80: malignant neoplasm, without specification of site |
| **Diagnosis List Two: Less Conservative** |
| Diagnoses from list one, plus |
| **ICD-10** |
| 80003: neoplasm, malignant |
| 80006: neoplasm, metastatic |
| 80103: carcinoma, NOS |
| 80106: carcinoma, metastatic, NOS |
| 81406: adenocarcinoma, metastatic, NOS |
| 84906: metastatic signet ring cell carcinoma |
| **Diagnosis List Three: Most Inclusive** |
| Diagnoses from list one and two, plus: |
| C00–C14 Malignant neoplasms, lip, oral cavity and pharynx |
| C30–C39 Malignant neoplasms, respiratory system and intrathoracic organs |
| C40–C41 Malignant neoplasms, bone and articular cartilage |
| C43–C44 Malignant neoplasms, skin |
| C45–C49 Malignant neoplasms, connective and soft tissue |
| C50–C58 Malignant neoplasms, breast and female genital organs |
| C60–C63 Malignant neoplasms of male genital organs |
| C64–C68 Malignant neoplasms, urinary organs |
| C69–C72 Malignant neoplasms, eye, brain and central nervous system |
| C73–C75 Malignant neoplasms, endocrine glands and related structures |
| C81–C96 Malignant neoplasms, stated or presumed to be primary, of lymphoid, haematopoietic and related tissue |
| C97 Malignant neoplasms of independent (primary) multiple sites |
| 140-149 Malignant neoplasm of lip, oral cavity, and pharynx |
| 160-165 Malignant neoplasm of respiratory and intrathoracic organs |
| 170-175 Malignant neoplasm of bone, connective tissue, skin, and breast |
| 179-189 Malignant neoplasm of genitourinary organs |
| 190-199 Malignant neoplasm of other and unspecified sites |
| 200-208 Malignant neoplasm of lymphatic and hematopoietic tissue |
| 209 Neuroendocrine tumors |
| 235-238 Neoplasms of uncertain behavior |
| 239 Neoplasms of unspecified nature |

ICD= international classification of disease
