# Supplementary material for: Validating an algorithm to identify metastatic gastric cancer in the absence of routinely collected TNM staging data
Source: BMC Health Serv Res. 2018 May 2;18:309. doi: 10.1186/s12913-018-3125-7 (PMC5930789; doi:10.1186/s12913-018-3125-7)
Supplement: Supplementary file 2 — Table S2. Algorithm properties when the patient cohort was restricted to those who received a surgical resection, Sensitivity, specificity, negative predictive value, positive predictive value and accuracy for the algorithms when applied to a subset of patients who received a surgical resection. (DOCX 15 kb) [file 12913_2018_3125_MOESM2_ESM.docx]

| Table S2: Algorithm properties when the patient cohort was restricted to those who received a surgical resection | | | | | |
| --- | --- | --- | --- | --- | --- |
| **Algorithm** | **Se (95% CI)** | **Sp (95%CI)** | **PPV (95%CI)** | **NPV (95%CI)** | **A (95% CI)** |
| **+/- 3 months** | | | | | |
| **Conservative Diagnostic Codes** | | | | | |
| 28. 1+ hospitalization | 33.8 (28.3-39.2) | 94.2 (92.3-95.9) | 72.3 (64.5-79.5) | 76 (73.0-78.8) | 75.4 (72.6-78.2) |
| 29. 1+ hospitalization or 2+ outpatient | 40 (34.3-45.5) | 89.1 (86.6-91.5) | 62.3 (55.2-69.1) | 76.7 (73.7-79.7) | 73.8 (71.0-76.7) |
| 30. 1+ hospitalization or 1+ outpatient | 43 (37.2-48.7) | 86.5 (83.8-89.2) | 58.9 (52.3-65.5) | 77.1 (74.0-80.1) | 73 (70.1-75.7) |
| **Less Conservative Diagnostic Codes** | | | | | |
| 31. 1+ hospitalization | 41.3 (35.7-47.0) | 88.5 (85.9-90.9) | 61.8 (55.1-68.4) | 77 (74.0-79.9) | 73.8 (71.0-76.6) |
| 32. 1+ hospitalization or 2+ outpatient | 47.1 (41.5-52.8) | 83.3 (80.4-86.1) | 55.9 (49.9-62.1) | 77.8 (74.6-80.8) | 72 (69.2-74.9) |
| 33. 1+ hospitalization or 1+ outpatient | 50.2 (44.5-55.9) | 80.9 (78.0-84.0) | 54.3 (48.5-60.1) | 78.3 (75.1-81.3) | 71.4 (68.5-74.4) |
| **Most Inclusive Diagnostic Codes** | | | | | |
| 34. 1+ hospitalization | 42.7 (37.1-48.4) | 87.3 (84.6-89.8) | 60.2 (53.6-66.7) | 77.2 (74.1-80.1) | 73.4 (70.6-76.2) |
| 35. 1+ hospitalization or 2+ outpatient | 57 (51.2-62.7) | 69.4 (65.9-72.9) | 45.6 (40.6-50.7) | 78.2 (74.7-81.5) | 65.6 (62.6-68.6) |
| 36. 1+ hospitalization or 1+ outpatient | 64.8 (59.3-70.1) | 59.3 (55.5-63.1) | 41.7 (37.1-46.3) | 78.9 (75.4-82.4) | 60.9 (57.9-64.1) |
| **+/- 6 months** | | | | | |
| **Conservative Diagnostic Codes** | | | | | |
| 37. 1+ hospitalization | 40.6 (35.0-46.4) | 92 (89.8-94.0) | 69.7 (62.7-76.3) | 77.5 (74.5-80.4) | 76.1 (73.3-78.8) |
| 38. 1+ hospitalization or 2+ outpatient | 47.4 (41.8-53.1) | 85.1 (82.4-87.8) | 59 (52.8-65.0) | 78.2 (75.2-81.3) | 73.4 (70.6-76.2) |
| 39. 1+ hospitalization or 1+ outpatient | 51.2 (45.6-56.9) | 82 (79.1-84.9) | 56.2 (50.4-62.1) | 78.9 (75.8-81.9) | 72.5 (69.6-75.2) |
| **Less Conservative Diagnostic Codes** | | | | | |
| 40. 1+ hospitalization | 46.8 (41.2-52.5) | 85 (82.2-87.6) | 58.4 (52.1-64.6) | 78 (74.9-80.9) | 73.2 (70.1-76.0) |
| 41. 1+ hospitalization or 2+ outpatient | 53 (47.3-58.6) | 78.3 (75.2-81.6) | 52.4 (46.8-58.0) | 78.7 (75.5-81.7) | 70.4 (67.6-73.4) |
| 42. 1+ hospitalization or 1+ outpatient | 56.7 (50.9-62.3) | 75.1 (71.8-78.5) | 50.6 (45.4-56.1) | 79.4 (76.2-82.4) | 69.4 (66.5-72.4) |
| **Most Inclusive Diagnostic Codes** | | | | | |
| 43. 1+ hospitalization | 48.5 (42.7-54.2) | 83.3 (80.4-86.1) | 56.6 (50.6-62.6) | 78.2 (75.1-81.2) | 72.5 (69.6-75.3) |
| 44. 1+ hospitalization or 2+ outpatient | 66.2 (60.8-71.6) | 58.5 (54.7-62.4) | 41.8 (37.4-46.3) | 79.4 (75.7-82.9) | 60.9 (57.8-64.0) |
| 45. 1+ hospitalization or 1+ outpatient | 73.1 (68.0-77.9) | 48.2 (44.5-52.1) | 38.8 (34.8-43.0) | 79.9 (76.0-83.7) | 55.9 (52.8-59.1) |
