# Supplementary material for: Validating an algorithm to identify metastatic gastric cancer in the absence of routinely collected TNM staging data
Source: BMC Health Serv Res. 2018 May 2;18:309. doi: 10.1186/s12913-018-3125-7 (PMC5930789; doi:10.1186/s12913-018-3125-7)
Supplement: Supplementary file 3 — Table S3. Number of patients in each cell, by algorithm, The breakdown of the number of true positive, true negatives, false positives and false negatives for each algorithm. (DOCX 19 kb) [file 12913_2018_3125_MOESM3_ESM.docx]

| **Table S3: Number of patients in each cell, by algorithm** | | |  |  |
| --- | --- | --- | --- | --- |
|  | **Number of patients** | | | |
| **Algorithm** | **+/+** | **+/-** | **-/+** | **-/-** |
| **+/- 3 Months Diagnosis Date** |  |  |  |  |
| **Conservative Diagnostic Codes** |  |  |  |  |
| 1.      1+ hospitalization | 642 | 81 | 643 | 1000 |
| 2.      1+ hospitalization or 2+ outpatient | 742 | 142 | 543 | 939 |
| 3.      1+ hospitalization or 1+ outpatient | 786 | 171 | 499 | 910 |
| **Less Conservative Diagnostic Codes** |  |  |  |  |
| 4.      1+ hospitalization | 679 | 131 | 606 | 950 |
| 5.      1+ hospitalization or 2+ outpatient | 776 | 193 | 509 | 888 |
| 6.      1+ hospitalization or 1+ outpatient | 824 | 219 | 461 | 862 |
| **Most Inclusive Diagnostic Codes** |  |  |  |  |
| 7.      1+ hospitalization | 688 | 142 | 597 | 939 |
| 8.      1+ hospitalization or 2+ outpatient | 892 | 346 | 393 | 735 |
| 9.      1+ hospitalization or 1+ outpatient | 979 | 446 | 306 | 635 |
| +/- **6 months Diagnosis Date** |  |  |  |  |
| **Conservative Diagnostic Codes** |  |  |  |  |
| 10.  1+ hospitalization | 739 | 107 | 546 | 974 |
| 11.  1+ hospitalization or 2+ outpatient | 845 | 191 | 440 | 890 |
| 12.  1+ hospitalization or 1+ outpatient | 881 | 226 | 404 | 855 |
| **Less Conservative Diagnostic Codes** |  |  |  |  |
| 13.  1+ hospitalization | 772 | 169 | 513 | 912 |
| 14.  1+ hospitalization or 2+ outpatient | 875 | 248 | 410 | 833 |
| 15.  1+ hospitalization or 1+ outpatient | 912 | 283 | 373 | 798 |
| **Most Inclusive Diagnostic Codes** |  |  |  |  |
| 16.  1+ hospitalization | 785 | 184 | 500 | 897 |
| 17.  1+ hospitalization or 2+ outpatient | 994 | 457 | 291 | 624 |
| 18.  1+ hospitalization or 1+ outpatient | 1062 | 560 | 223 | 521 |
| **-3 months Diagnosis, No Time Limit** |  |  |  |  |
| **Conservative Diagnostic Codes** |  |  |  |  |
| 19.  1+ hospitalization | 908 | 326 | 337 | 755 |
| 20.  1+ hospitalization or 2+ outpatient | 994 | 428 | 291 | 653 |
| 21.  1+ hospitalization or 1+ outpatient | 1030 | 480 | 255 | 601 |
| **Less Conservative Diagnostic Codes** |  |  |  |  |
| 22.  1+ hospitalization | 937 | 390 | 348 | 691 |
| 23.  1+ hospitalization or 2+ outpatient | 1019 | 486 | 266 | 595 |
| 24.  1+ hospitalization or 1+ outpatient | 1056 | 536 | 229 | 545 |
| **Most Inclusive Diagnostic Codes** |  |  |  |  |
| 25.  1+ hospitalization | 951 | 411 | 334 | 670 |
| 26.  1+ hospitalization or 2+ outpatient | 1112 | 699 | 173 | 382 |
| 27.  1+ hospitalization or 1+ outpatient | 1157 | 783 | 128 | 298 |
| **Limited to Patients with a Surgical Resection** |  |  |  |  |
| **+/- 3 months from diagnosis** |  |  |  |  |
| Conservative Diagnostic Codes |  |  |  |  |
| 28.  1+ hospitalization | 99 | 38 | 194 | 613 |
| 29.  1+ hospitalization or 2+ outpatient | 117 | 71 | 176 | 580 |
| 30.  1+ hospitalization or 1+ outpatient | 126 | 88 | 167 | 563 |
| Less Conservative Diagnostic Codes |  |  |  |  |
| 31.  1+ hospitalization | 121 | 75 | 172 | 576 |
| 32.  1+ hospitalization or 2+ outpatient | 138 | 109 | 155 | 542 |
| 33.  1+ hospitalization or 1+ outpatient | 147 | 124 | 146 | 527 |
| Most Inclusive Diagnostic Codes |  |  |  |  |
| 34.  1+ hospitalization | 125 | 83 | 168 | 568 |
| 35.  1+ hospitalization or 2+ outpatient | 167 | 199 | 126 | 452 |
| 36.  1+ hospitalization or 1+ outpatient | 190 | 265 | 103 | 386 |
| **Limited to Patients with a Surgical Resection** |  |  |  |  |
| **+/- 6 months from diagnosis** |  |  |  |  |
| **Conservative Diagnostic Codes** |  |  |  |  |
| 37.  1+ hospitalization | 119 | 52 | 174 | 599 |
| 38.  1+ hospitalization or 2+ outpatient | 139 | 97 | 154 | 554 |
| 39.  1+ hospitalization or 1+ outpatient | 150 | 117 | 143 | 534 |
| **Less Conservative Diagnostic Codes** |  |  |  |  |
| 40.  1+ hospitalization | 137 | 98 | 156 | 553 |
| 41.  1+ hospitalization or 2+ outpatient | 155 | 141 | 138 | 510 |
| 42.  1+ hospitalization or 1+ outpatient | 166 | 162 | 127 | 489 |
| **Most Inclusive Diagnostic Codes** |  |  |  |  |
| 43.  1+ hospitalization | 142 | 109 | 151 | 542 |
| 44.  1+ hospitalization or 2+ outpatient | 194 | 270 | 99 | 381 |
| 45.  1+ hospitalization or 1+ outpatient | 214 | 337 | 79 | 314 |
| +/-: test positive, true positive; +/- test positive, true negative; -/+: test negative, true positive; -/-: test negative, true negative | | | | |
